# Protective Effect of Kaempferol and Its Nanoparticles on 5-Fluorouracil-Induced Cardiotoxicity in Rats

**DOI:** 10.1155/2022/2273000

**Published:** 2022-02-13

**Authors:** Soheila Safarpour, Marzieh Pirzadeh, Anahita Ebrahimpour, Fatemeh Shirafkan, Fateme Madani, Mohammad Hosseini, Ali Akbar Moghadamnia, Sohrab Kazemi

**Affiliations:** ^1^Student Research Committee, Babol University of Medical Sciences, Babol, Iran; ^2^Department of Pharmacology and Toxicology, School of Medicine, Babol University of Medical Sciences, Babol, Iran; ^3^Cellular and Molecular Biology Research Center, Health Research Institute, Babol University of Medical Sciences, Babol, Iran; ^4^Department of Veterinary Pathology, Babol Branch, Islamic Azad University, Babol, Iran

## Abstract

**Background:**

Fluorouracil (5-FU) is the third most common chemotherapeutic agent used in the treatment of solid tumors. 5-FU-associated cardiotoxicity ranks the second causes of cardiotoxicity induced by chemotherapeutic drugs after anthracyclines. Kaempferol (KPF), a common flavonoid, possessing anti-inflammatory, antiapoptotic, antioxidative properties, and its protective effects on cardiovascular disease has been reported in various studies. The current study is aimed at appraising the effect of KPF and KPF nanoparticles (NPs) on 5-FU-induced cardiotoxicity in rats.

**Methods:**

Thirty Male Wistar rats were divided into five groups as follows: control, 5-FU, 5-FU+10 mg/kg vitamin C, 5-FU+ 1 mg/kg KPF, and 5-FU+ 1 mg/kg KPF-NPs. Cardiotoxicity was induced with an intraperitoneal injection of a single dose of 5-FU (100 mg/kg). The control group received normal saline, and the treatment groups received KPF and KPF-NPs with an intraperitoneal injection for 14 days. Each heart histopathological lesions were given a score of 0 to 3 in compliance with the articles for statistical analysis.

**Results:**

5-FU resulted in a significant cardiotoxicity represented by an increase in cardiac enzymes, MDA (malondialdehyde) levels, COX-2 (cyclooxygenase-2) expression, and histopathological degenerations. 5-FU treatment also decreased body weight, TAC (total antioxidant capacity) values, VEGF (vascular endothelial growth factor) expression, blood cells, and hemoglobin (Hb) levels. Treatment with KPF and KPF-NPs reduced oxidative stress, cardiac enzymes, COX-2 expression, and VEGF expression. The number of blood cells, Hb levels, and histopathological degenerations, in cardiac tissue also body weight of animals, increased, followed by treatment with KPF and KPF-NPs.

**Conclusion:**

Our results demonstrated that treatment with KPF and KPF-NPs significantly improved cardiotoxicity induced by 5-FU in rats.

## 1. Introduction

The fluoropyrimidine 5-fluorouracil (5-FU) is an antimetabolite drug used as the third most common chemotherapeutic agent to cure cancers, particularly colorectal, skin, and breast cancers [1, 2].

5-FU anticancer effects are applied by inhibition of thymidylate synthase enzyme and incorporation its metabolites into RNA and DNA [3, 4]. Treatment with 5-FU increases the fraction of ribosome-free L11, L5, and L23 ribosomal proteins and their interaction with MDM2 (Mouse double minute 2 homolog), leading to activation of p53 protein and G1/S arrest. 5-FU has also been shown to induce apoptosis of cancer cells by suppressing NF-*κβ* factor activity, leading to the activation of the proapoptotic pathway [5].

Despite the benefits of fluoropyrimidine, it may provoke various side effects such as nausea, mucositis, emesis, myelosuppression, and toxic heart reactions, and the risks and toxicities associated with this drug should be measured [6].

5-FU is the second most current drug associated with cardiac toxicity [7]. The most common manifestation of fluoropyrimidine-induced cardiac toxicity such as 5-FU is unusual chest pain, which is seen as angina during stress or rest tolerance, and acute coronary syndrome [8–10]. Autoimmune-mediated injury of the myocardium, coronary artery spasm, damage to the endothelium, global dysfunction, thrombogenic effects, reposition of metabolites, and direct toxic effects on myocardium causing necrosis are the possible mechanisms proposed for cardiotoxicity induced by 5-FU. Several signaling pathways, including MAPK/ERK1/ROS, are involved in 5-FU-induced heart damage. One of the known injuries of 5-FU is vasospasm due to endothelial dysfunction, and an essential signaling pathway in causing this damage is associated with COX-2, which alone or under the influence of ROS, leads to vasospasm [11] It is known that species derived from oxygen are cytotoxic. One of the effects of oxidative damage to cellular DNA is mutation, including DNA sequence rearrangement and gene amplification which happens in the first stage of cancers. Free radical-induced DNA damage was detected in the various cancer tissues [12]. Oxidative stress is considered to be the main cause of colorectal cancer pathogenesis. Hydrogen peroxide and superoxide radicals are involved in the anticancer mechanism of 5-FU and also responsible for the various side effects of such therapies. Oxidative stress in chemotherapy-treated cancer patients was evidenced by an increase in blood lipid peroxidation and a decrease in plasma antioxidant levels [13].

However, the pathology and its specific mechanisms have not yet been fully elucidated [14, 15]. Different mechanisms including direct drug or drug metabolite of 5-FU induced cardiotoxicity are proposed. The pathogenesis of 5-FU-induced cardiotoxicity may involve cellular damage due to the oxidative stress and the induction of apoptosis [16]. Previous studies showed that 5-FU could cause interstitial fibrosis and inflammatory reactions in the myocardium; platelet aggregation due to damage of the arterial endothelium; hemorrhagic infarction, increased myocardial energy metabolism, vasoconstriction of arteries; reduced antioxidant capacity and increased superoxide anion levels; and changes in RBC structure and metabolism [17]. Antioxidant activity such as glutathione peroxidase and superoxide dismutase was diminished followed by 5-FU treatment in guinea pig [7].

Herbal medicines can be considered to protect against the toxic effects of chemotherapy drugs [18, 19]. Polyphenols are compounds composed mostly of phenolic acids and flavonoids, serving significant antiviral, antineoplastic, and antioxidant purposes and can be used to cure chronic diseases, including cardiovascular disease [20]. Kaempferol (3,4′,5,7-tetrahydroxyflavone), a flavonoid compound, is one of the most popular diet flavonoids in phytoestrogen, grapefruit, tea, broccoli, and plant sources [21]. Cardioprotective, antioxidant, antihyperglycemic, anti-inflammatory, and antiapoptotic effects of KPF have been reported in various studies. KPF treatment attenuated ischemia/reperfusion of cardiac injury. Furthermore, KPF protective effects on doxorubicin-induced cardiotoxicity both in vitro and in vivo have been also reported [22, 23]. However, their biological activity is limited due to the low absorption of these substances in the body. Among the causes of low adsorption and their bioavailability which have poor stability, low solubility in water, and inactive release of these substances, hence, the NP delivery system is already widely used in the pharmaceutical field to increase the absorption of active compounds [24].

Therefore, our study is aimed at investigating KPF's protective role on 5-FU-induced cardiotoxicity in male rats using pathological, biochemical, and molecular pathway changes of VEGF and COX-2 in rat heart tissues.

## 2. Materials and Methods

### 2.1. Chemicals

KPF powder (90%) was prepared from Sigma-Aldrich (CAS.N 520183). We obtained 5-FU from Sigma, St. Louis, MO (USA). We prepared sodium tripolyphosphate (STPP) from Dae-Jung (Korea). We also bought chitosan (CS), pentylenetetrazol (PTZ), and alginic acid sodium salt (ALG), Sigma-Aldrich (St. Louis, Mo). Total antioxidant capacity (TAC) and malondialdehyde (MDA) kits were prepared from the Company of Teb Pajohan Razi (Iran). RNA extraction kit and (Sinacolon, Tehran, Iran) cDNA synthesis kit were bought from Sinacolon and Yekta Tajhiz Azma Company, respectively (Tehran, Iran).

### 2.2. Preparation and Characterization of KPF-Loaded NPs

The CS, ALG, and STPP solutions have been sonicated to prepare KPF-loaded NPs according to the study of Ahmadi et al. [25]. In brief, 10 ml of 1% acetic acid was used for dissolving 10 mg of CS, and it was stirred via ultrasonic irradiation (Bandelin, Germany) for an hour. We dissolved 1 mg of KPF powder in 1 ml absolute ethanol and stepped up gradually in a dropwise manner to the solution of CS. Moreover, 5 mg ALG in 5 ml distilled water and STPP (0.13% w/v) was added to the solution and centrifuged at 14000 rpm for 30 min.

#### 2.2.1. Particle Size and Loading Content

The distribution of KPF-NPs and the scattering index (PDI) were measured using the light scattering technique (DLS) Nano-ZS ZEN 3600 (Malvern Instruments, Ltd., UK).

#### 2.2.2. Nanoparticle Morphology

Scanning electron microscopy (SEM) (Quanta FEG 250; FEI, North America) was employed for the surface morphology of the KPF-loaded chitosan NPs [26].

#### 2.2.3. HPLC Analysis of KPF

The obtained pellet was dissolved in deionized water and utilized for in vivo tests. In brief, 1 mg of KPF or an equivalent of KPF-loaded NPs was blended with 1 ml of deionized water, and the mixture was vortexed for 5 min. Afterward, the combination was centrifuged for 5 min at 20000 rpm. Separating KPF from the supernatant, an ethyl acetate solution (1 : 1, v/v) was used to separate the KPF extract from the supernatant solution. The high-performance liquid chromatography at 420 nm wavelength was used for the measurement of KPF concentration.

### 2.3. Animals

Thirty male Wistar rats (200-250 g) from the animal place of Babol University of Medical Sciences (Babol, Iran) were kept in a 12 h light/dark situation with free admittance to water and food. All trial methods were approbated via the Babol University of Medical Sciences ethical committee (IR.MUBABOL.HRI.REC.1398.252).

### 2.4. Experimental Groups

**Figure 1:**
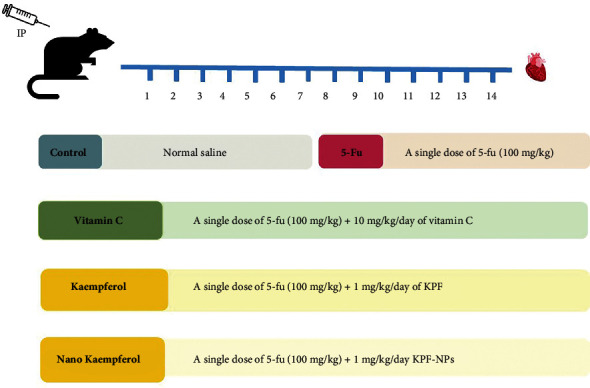
Rats were divided into five groups and were injected IP for 14 days. Control group: animals received normal saline. The 5-FU group: animals received IP injection of a single dose of 5-FU (100 mg/kg) only on the first day. The vitamin C group: the group received 5-FU at a single dose of 100 mg/kg body weight only on the first day of treatment and from day 2 onwards alone, rats were injected with 10 mg/kg/day of vitamin C for 14 days. The KPF treatment group: animals received a single dose of 5-FU (100 mg/kg) only on the first day of treatment and from day 2 onwards alone, rats were injected with 1 mg/kg/day of KPF for 14 days. The KPF-NP treatment group: animals received a single dose of 5-FU (100 mg/kg) only on the first day of treatment and from day 2 onwards alone, rats were injected with 1 mg/kg/day of KPF-NPs for 14 days. The hearts of the animals were then analyzed (number of animals in each group: 6).

The animals were injected intraperitoneally (IP) every day for two weeks (Figure 1) [27].

Thirty male Wistar rats were randomly divided into five groups as follows:

1. Normal group (*n* = 6): animals received normal saline (phosphate-buffered saline) for 14 days
2. The 5-FU group (*n* = 6): animals received IP injection of a single dose of 5-FU (100 mg/kg) only on the first day of treatment, and no 5-FU injection was performed from the second day [16, 28]
3. The positive control group (*n* = 6): the group received 5-FU at a single dose of 100 mg/kg body weight only on the first day of treatment, and no 5-FU injection was performed from the second day. From day 2 onwards alone, rats were injected with 10 mg/kg/day of vitamin C as IP for 14 days [29]
4. The KPF treatment group (*n* = 6): animals received IP injection of a single dose of 5-FU (100 mg/kg) only on the first day of treatment, and no 5-FU injection was performed from the second day. From day 2 onwards alone, rats were injected with 1 mg/kg/day of KPF as IP for 14 days [30]
5. The KPF-NPs treatment group (*n* = 6): in this group, animals received IP injection of a single dose of 5-FU (100 mg/kg) only on the first day of treatment, and no 5-FU injection was performed from the second day. From day 2 onwards alone, rats were injected with 1 mg/kg/day of KPF-NPs as IP for 14 days

The rats in all groups were weighed before and after injection. Animals were then sacrificed, and their blood was collected for further analysis. Heart tissues were also removed for evaluation of biochemical, molecular, and histopathological analysis.

The rats in all groups were weighed before and after injection. Animals were then sacrificed, and their blood was collected for further analysis. Heart tissues were also removed for evaluation of biochemical, molecular, and histopathological analysis.

### 2.5. Blood Collection

Rats were anesthetized with an ether overdose; a heart puncture was performed to collect blood samples. White blood cells (WBC), red blood cells (RBC), and platelets (PLT) were counted, and hemoglobin (Hb) levels were measured. The total number of blood cells and Hb levels was specified via a Neubauer chamber in blood samples diluted in Turk's solution (1 : 10). Also, the levels of cardiac enzymes including aspartate transaminase (AST), lactate dehydrogenase (LDH), and creatine kinase myocardial band (CK-MB) were evaluated. The serum was separated through centrifugation of blood at 14°C, 2500 rpm, and 15 min for further analysis.

### 2.6. Total Antioxidant Capacity Assay (TAC)

In brief, after anesthetizing the animal and blood collection, heart tissues were separated and immediately transferred to a microtube, and then all heart tissue samples were stored in -80°C until evaluating the biochemical indexes. Every heart tissue was also well balanced in normal saline with a volume of 0.5 *μ*L and after centrifugation at 1000 g for 5 minutes, the soups were used for the analysis of biochemical parameters with TAC kit. We added 1.5 ml of ready-to-use FRAP reagent to all tubes and incubated them at 5°C for 37 minutes. Afterward, we added 51 *μ*L of the sample (tissue extracts or different standards) to the tubes and blended them well, and the mixtures were reincubated at 37°C for 15 minutes. Then, color intensity was measured by ELISA reader at the wavelength of 593 nm in front of the blank (1.5 ml of FRAP solution plus 50 *μ*L of distilled water) [31].

### 2.7. Malondialdehyde Test (MDA)

The MDA assay has been used for the measurement of oxidative stress levels in the heart tissue. To measure MDA levels, 1 ml of tissue was combined with 2 ml of MDA reagent and posited in a boiling water bath for an hour. After incubating of the samples in a 10-minute ice bath, they were centrifuged for another 10 minutes at 2500 rpm. With MDA kit, thiobarbituric acid will react with MDA to form a red product. The light uptake was measured via ELISA reader at 535 nm [32].

### 2.8. Histopathological Evaluation of the Heart

We collected heart samples and fixed them in 4% paraformaldehyde. After that, the samples were drowned in paraffin, and a microtome instrument (model Leitz 1512, Germany) was utilized to prepare the sections (6 *μ*m).

#### 2.8.1. Hematoxylin-Eosin Staining

Hematoxylin-eosin staining was used for measuring the histopathological degeneration in the heart tissue. For each group, five animals, from each animal, seven slides, in each slide, three fields were selected, and the amount of inflammation was estimated. For histological studies, data analysis was performed by an expert pathologist using ImageJ software.

Briefly, the coloring steps are as follows: placing tissue incisions in 100% alcohol (five minutes), placing tissue incisions in 96% alcohol (five minutes), staining with hematoxylin (five minutes), rinsing with running water (five minutes), eosin staining (15 seconds), immerse in distilled water for decolorization, ethanol 70% (15 seconds), ethanol 95% (30 seconds), absolute ethanol (one minute), xylene (five minutes), and paste with entellan.

### 2.9. RNA Extraction and Real-Time Polymerase Chain Reaction (RT-PCR)

In the following, for evaluation of VEGF and COX-2 genes, the remaining heart samples were immediately transferred to RNA later, the samples were stored in a -20 freezer overnight, then the liquid was removed, and all tissues transferred to -80 freezer and stored until RNA extraction.

We extracted total RNA based on the total RNA extraction kit protocol (Pars Tous, Mashhad, Iran), and the cDNA was synthesized based on the manufacturer's protocol (Pars Tous, Mashhad, Iran). qRTPCR was accomplished using an ABI Step one plus real-time PCR system (Applied Biosystem, USA) with the primers set for COX-2 and VEGF as target genes and GAPDH as the housekeeping gene. 10 *μ*L real-time PCR reaction mixture consisted of 1 *μ*L cDNA, 6.25 *μ*L SYBR-Green (Amplicon high Rox master mix, Denmark), 2.25 *μ*L nuclease-free water, and 0.25 *μ*L of 10 pmol of each primer (Robin Teb Gostar, Tehran, Iran).

According to the study of Kirkpatrick et al. 2018 [33], conditions for the reverse transcription step were 25°C for 10 min, 37°C for 60 min, and 85°C for 5 min. The polymerase chain reaction was carried out by holding temperature for 15 min at 95°C and 40 cycles of 15 s at 95°C and 30 s at 62°C and 30 s at 72°C followed via melting curve temperature steps. The COX-2 and VEGF primers are represented in Table 1.

### 2.10. Statistical Analysis

We analyzed data of the current study as mean ± SD with GraphPad Prism software version 8. We performed a one-way analysis of variance (ANOVA) followed by Tukey posttest to analyze blood count results, biochemical assays, and Fisher's LSD posttest for molecular data. *P* values less than 0.05 were considered statistically significant.

## 3. Result

### 3.1. Particle Size and Nanoparticle Morphology

**Figure 2:**
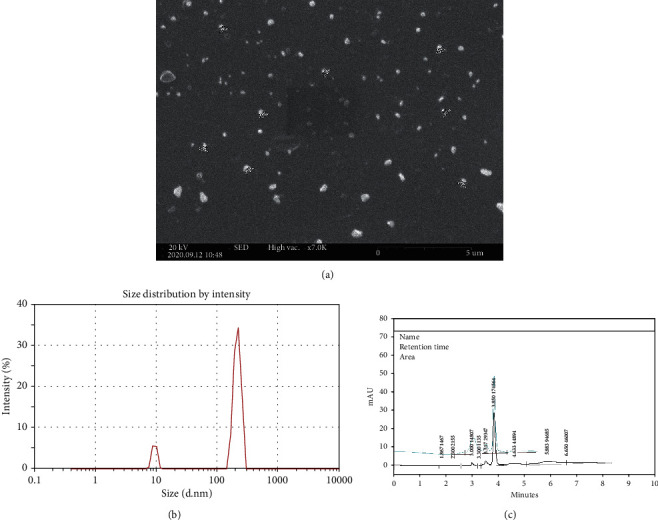
(a) Scanning electron microscopy images. Morphology of KPF-NPs. (b) 88.2% of the nanoparticles are in the first peak and have 212.6 nm. 11.8% have a size of 9.365 nm, which is seen in the second peak. (b) The analysis of HPLC. Curves of 10 ppm standard and 40 ppm KPF loaded Cs-STPP NPs.

The shape, surface texture, and size of KPF-NPs were detected with SEM, and its micrograph has been illustrated (Figure 2(a)).

DLS method was used to examine the average size of nanoparticles, and it has been shown that the average size of KPF-NPs is below 212.6 nm (Figure 2(b)). The polydispersity index (PDI), zeta potential, and the particle size of the KPF- NPs were determined by dynamic light scattering (Zetasizer Nano ZS, Malvern Instruments, UK) at 25°C.

By analyzing various concentrations of KPF-NPs in the peak area, the calibration curve was provided, and the concentration of KPF was measured by a regression equation created by a calibration curve. HPLC analysis indicates that the release efficiency of KPF-loaded CTS-STPP NPs was about 25% (Figure 2(c)).

### 3.2. Bodyweight

**Figure 3:**
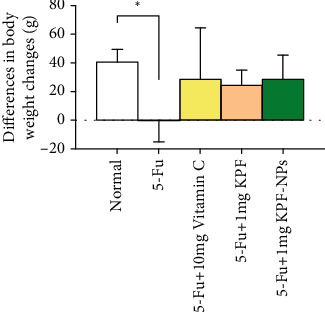
Comparison of the body weight changes in rats of different groups on the first day before injection and the 14th day after injection. All results are expressed as mean ± SD. ^∗^*P* < 0.05 significant compared to the normal group. SD: standard deviation (number of animals in each group: 6).

Bodyweight was measured on the first day of injection and the last day of injection, on the 14th day. Changes in body weight were measured and compared between the treated and control groups. The results showed that 5-FU inhibited weight gain compared to the normal group significantly (*P* < 0.05), which indicates growth retardation. However, KPF treatment increased the body weight of animals, and this increase was not significantly manner compared to the group receiving 5-FU (Figure 3).

### 3.3. The Effect of KPF, KPF-NPs, and 5-FU on Blood Cell Count and Cardiac Enzymes

Administration of 5-FU decreased the number of WBCs, RBCs, and platelets (*P* < 0.001) in a significant manner. As expected, RBC levels were remarkably higher in the group receiving KPF-NPs with doses of 1 mg/kg (*P* < 0.01) than in the 5-FU group. In addition, the group receiving KPF with the dose of 1 mg/kg demonstrated a remarkable increase in RBC count compared to the 5-FU group (*P* < 0.01).

**Figure 4:**
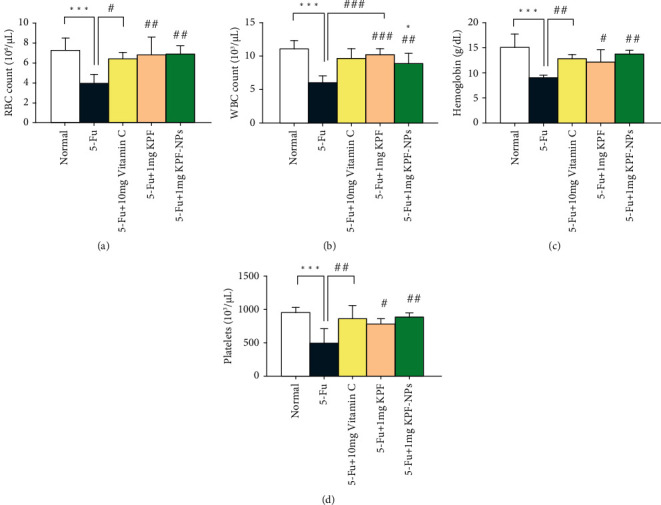
Data quantification indicates the effect of treated groups on blood factors and the comparison of different groups with each other. ^∗^*P* < 0.05, ^∗∗∗^*P* < 0.001 significantly compared with the normal group.^.#^*P* < 0.05, ^##^*P* < 0.01, ^###^*P* < 0.001 significantly compared to the 5-FU group. All results are presented as mean ± standard deviation. RBC: red blood cell; WBC: white blood cell (number of animals: 6).

Similarly, in the group receiving vitamin C, (positive control group), a remarkable increase in RBC count was noticed than in the 5-FU group (*P* < 0.05) (Figure 4(a)).

Regarding WBC count, administration of KPF-NPs decreased the number of WBCs (*P* < 0.05) in a significant manner compared to a normal group. On the other hand, the results showed a significant increase in the groups treated with KPF-NPs when compared to the group receiving 5-FU (*P* < 0.01). Moreover, treatment with KPF increased WBC count compared to the 5-FU group (*P* < 0.001). Likewise, the group receiving vitamin C demonstrated a significant difference in WBC count compared with the 5-FU group (*P* < 0.001) (Figure 4(b)).

Our study showed that Hb levels were significantly decreased in the 5-FU receiving group (*P* < 0.001). Treatment with KPF, KPF-NPs, and vitamin C increased Hb levels. However, this increase was significant in the groups treated with 1 mg/kg KPF (*P* < 0.05), 10 mg/kg vitamin C (*P* < 0.01), and 1 mg/kg KPF-NPs (*P* < 0.01) (Figure 4(c)).

We also observed that PLT count increased remarkably in the groups treated with KPF-NPs and vitamin C (*P* < 0.01) compared to the 5-FU receiving group. Furthermore, the group treated with 1 mg/kg KPF also showed a significant increase in PLT count than the 5-FU treated group (*P* < 0.05) (Figure 4).

### 3.4. The Effect of KPF, KPF-NPs, and 5-FU on Cardiac Enzymes

**Figure 5:**
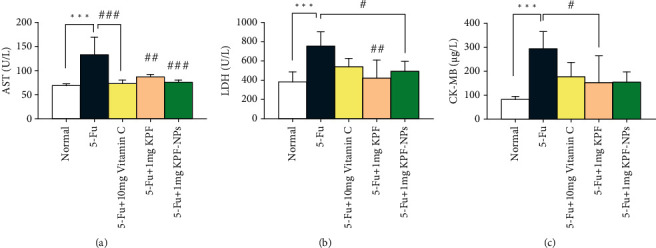
Data quantification indicates the effect of treated groups on cardiac enzyme levels and the comparison of different groups with each other. ^∗∗∗^*P* < 0.001 significantly compared to the normal group.^.#^*P* < 0.05, ^##^*P* < 0.01, ^###^*P* < 0.001 significantly compared to the 5-FU group. Results are presented as mean ± standard deviation. AST: aspartate transaminase; LDH: lactate dehydrogenase; CK-MB: creatine kinase-MB (number of animals: 6).

5-FU administration also increased cardiac enzyme levels including AST, LDH, and CK-MB compared to the normal group meaningfully (*P* < 0.001) (Figures 5(a)–5(c)).

Regarding AST enzyme, our results showed that AST serum levels were remarkably diminished in the group receiving 1 mg/kg of KPF-NPs (*P* < 0.001), the KPF treated (*P* < 0.01), and the positive control group (*P* < 0.001) compared to the 5-FU group (Figure 5(a)).

We also noticed a significant decrease in the serum levels of LDH enzyme in the group receiving KPF-NPs (*P* < 0.05) and also in the KPF treated group (*P* < 0.01) compared to the 5-FU group (Figure 5(b)).

In addition, in the CK enzyme which is a specific enzyme of the heart, a significant difference was found between the KPF group and the 5-FU group (*P* < 0.05) (Figure 5(c)).

### 3.5. The Effect of KPF, KPF-NPs, and 5-FU on Total Antioxidant Capacity and Oxidative Stress Values

**Figure 6:**
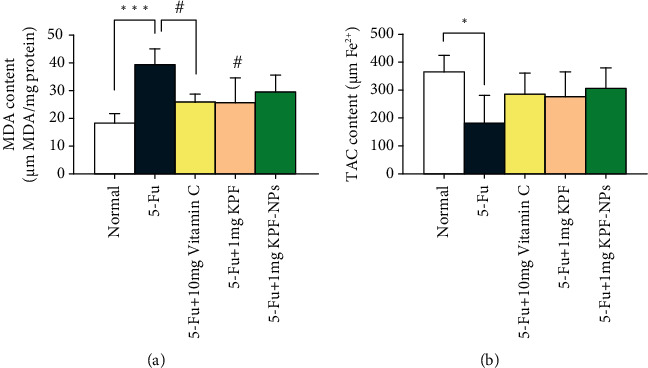
The effect of KPF and KPF-NP administration on oxidative stress values and total antioxidant capacity and comparison of different groups with each other. ^∗^*P* < 0.05, ^∗∗∗^*P* < 0.001 significantly compared with the normal group. ^#^*P* < 0.05 significantly compared to the 5-FU group. All results are presented as mean ± SD. MDA: malondialdehyde; TAC: total antioxidant capacity; SD: standard deviation (number of animals: 6).

MDA levels were elevated in the rats injected with 5-FU compared to the normal group meaningfully (*P* < 0.001). However, MDA levels were lessened in all treated groups, and only a significant reduction happened in KPF and vitamin C-treated groups (*P* < 0.05) (Figure 6(a)).

Administration of 5-FU significantly decreased TAC values compared to the normal group (*P* < 0.05). Treatment with KPF and KPF-NPs enhanced TAC values compared to the 5-FU group; however, this difference was not statistically significant (Figure 6(b)).

### 3.6. Effects of KPF, KPF-NPs, and 5-FU on Histopathological Changes of Heart Tissue

**Figure 7:**
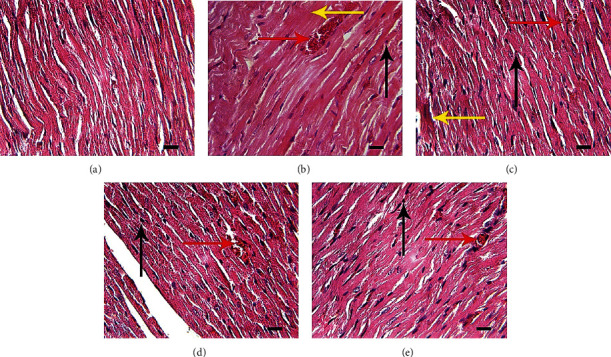
Histopathological changes of heart tissue. (a) Normal group: normal tissue conditions. (b) 5-FU group: hyperemia (right arrow), necrosis (up arrow), and hyaline (left arrow). (c) 5-FU group and 10 mg vitamin: hyperemia (right arrow), necrosis (up arrow), and hyaline (left arrow). (d) 5-FU and 1 mg/kg KPF-NP group: necrosis (up arrow). (e) 5-FU and 1 mg/kg KPF group: hyperemia (right arrow), necrosis (flash up) above, H&E coloring, and magnification: ×40 (bar = 100 *μ*m).

H&E staining results demonstrated heart cells' morphology was normal in the control group (Figure 7(a)). On the other hand, the 5-FU group that received no protection showed high levels of cardiac intoxication and prominent histopathological abnormalities, including hyaline formation, necrosis, and hyperemia (Figure 7(b)). However, the vitamin C group showed less damage in terms of hyperemia and necrosis (Figure 7(c)). The groups treated with KPF and KPF-NPs showed less tissue damage than the 5-FU group. Among other things, the group receiving KPF-NPs with a dose of 1 mg/kg (Figure 7(d)) and also the KPF group (Figure 7(e)) had no hyaline formation and also showed necrosis and hyperemia at a lower level than the 5-FU group. This indicates the improvement of damage and tissue abnormalities by KPF and KPF-NPs (Table 2).

### 3.7. The Effect of KPF, KPF-NPs, and 5-FU on COX-2 and VEGF Angiogenic Factors

**Figure 8:**
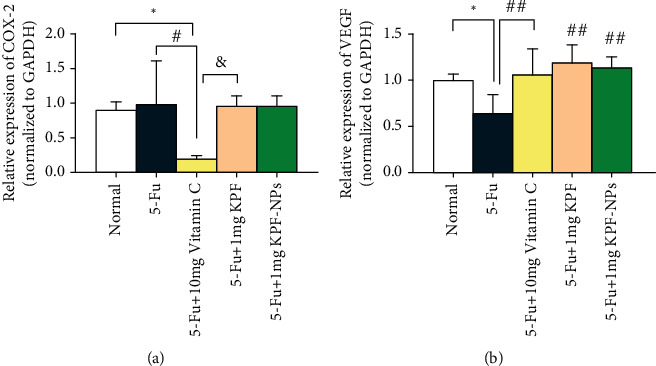
The effect of KPF and KPF-NP administration on the expression of genes related to angiogenesis such as COX-2 and VEGF and then comparison of different groups with each other. ^∗^*P* < 0.05 significantly compared to the normal group. ^#^*P* < 0.05, ^##^*P* < 0.01 significantly compared to the 5-FU group. ^&^*P* < 0.05 significant comparison between the 5-FU+10 mg vitamin C group and 5-FU+ 1 mg KPF group. All results are expressed as mean ± SD. COX: cyclooxygenase; VEGF: *v*ascular endothelial growth factor; SD: standard deviation (number of animals: 6).

In the case of the COX-2 gene expression, the results showed a significant reduction in COX-2 gene expression in the vitamin C group compared with the normal group (*P* < 0.05) and also compared to the 5-FU group (*P* < 0.05). Another significant discrepancy was also noticed between the vitamin C group and the KPF group (*P* < 0.05) (Figure 8(a)). Results also demonstrated that KPF-NPs decreased COX-2 gene expression compared to the 5-FU group but without any significant difference.

In the case of the VEGF gene, the expression of this gene was diminished in animals treated with 5-FU compared with the normal group (*P* < 0.05). In addition, expression of this gene was significantly enhanced in animals receiving KPF (*P* < 0.01), KPF-NPs (*P* < 0.01), and the vitamin C group (*P* < 0.01) than the 5-FU group (Figure 8(b)).

## 4. Discussion

Generally, the results of the current study showed that KPF and its NPs can increase RBC, WBC, HB, and PLT, as well as a reduction in AST, LDH, CK enzymes, and oxidative stress and in this way to protect the heart tissue and reduce damage and abnormalities to the heart, including necrosis, hyperemia and hyaline.

At present, one of the basic methods for cancer treatment is chemotherapy [34]. However, one of the main problems of this method is its associated side effects, including tissue damage such as intestinal mucositis, damage to the spleen and liver, and heart damage [35].

In the present study, rats treated with 5-FU demonstrated a significant decrease in body weight compared to the control group. According to previous studies, 5-FU treated rats showed a reduction in food intake, and a significant reduction in body weight which could be due to the damage to internal organs such as the liver [36] or intestine and a decrease in anaerobic bacteria in the gut [37]. However, bodyweight increased in treatment groups when compared to the 5-FU receiving group, but the difference was not statistically significant (Figure 3). Similarly, it has been shown that KPF pretreatment (1–10 mg/kg IP, before DOX administration) had no significant effect on improving the bodyweight of doxorubicin-induced myocardial damage in rats [21]. In contrast, another study showed the significant protective effect of KPF (21 mg/kg body weight) on weight loss caused by 3-nitropropionic acid [38].

Anemia is one of the most usual side effects of chemotherapy drugs, and continuous administration of single-agent 5-FU can cause about 50% of first- and second-degree anemias [39, 40].

Because RBCs have a high content of polyunsaturated fatty acids and high levels of HB, they can easily be exposed to oxidative damage and can be used as a model for examining oxidative damage in biological membranes. On the other hand, erythrocyte lipid peroxidation can be associated with cell aging. Therefore, it makes sense to suggest the use of antioxidants such as KPF in foods to protect blood cells from oxidative damage caused by free radical-related diseases or the use of certain drugs [41]. Other study findings in this field showed moderate thrombocytopenia at 7 days after intraperitoneal administration of 5-FU (150 mg/kg) and stable reversible thrombocytosis from 11 to 17 days after 5-FU injection. In fact, increased PLT production after 5-FU is associated with concomitant stimulation of the megakaryocyte-producing chamber in the rat spleen [42]. The 5-FU can also cause leukopenia, which is relieved by a glutamine-containing diet [43].

Following the findings mentioned above and in line with our previous study [44], we found that WBC, RBC, and PLT counts and Hb levels in the 5-FU group were significantly reduced. However, KPF-treated groups, as well as KPF-NPs, significantly prevented the reduction of RBC, WBC, and PLT counts as well as Hb levels induced the 5-FU.

KPF can also have a protective effect on heart tissue by lowering blood glucose levels, reducing oxidative stress, inflammation, apoptosis, and cardiac injury markers [45]. Also, KPF can show a significant level of protection against RBC, which may be because of its antioxidant property and ability to scavenge free radicals, thus preventing oxidative damage to RBC membranes [46].

The present results showed that administration of 5-FU increased AST, LDH, and CK-MB levels. Because these enzymes are so abundant in the heart, they can be used as a momentous indicator to identify heart damage. We also found that KPF and KPF-NPs protect heart tissue against 5-FU-induced cardiac toxicity represented by a significant reduction in AST, LDH, and CK-MB plasma levels.

In line with the present study, a study showed that 5-FU injection increased AST, alanine aminotransferase (ALT), and CK in 5-FU-induced cardiotoxicity in rats [44, 47]. It can be said that an increase in serum cardiac enzymes can be due to the damage to myocardial cells which ultimately increases the leakage of these serum enzymes. In contrast, treatment with KPF (intraperitoneally for a period of 7 days at different doses 1, 3, and 10 mg/kg body) can significantly decrease serum LDH, CK-MB, and troponin-I [30]. Thus, KPF treatment may increase cell integrity and reduce cell damage, which in turn reduces serum cardiac enzymes [27]. The results of the present study are in line with the reports of previous studies on the reduction of LDH and CK after KPF treatment in rats with myocardial infarction [48]. In addition, KPF treatment improved diastolic and systolic blood pressure [45].

The leading cause of 5-FU-induced cardiotoxicity is not fully understood [16]. Another hypothesis for cardiac toxicity of 5-FU is oxidative stress [49]. Oxidative stress is a disturbance in the balance between removal and production of ROS. ROS can cause modifications and irreversible damage to proteins, nucleic acids, and macromolecules [50], leading to stimulation of inflammatory mechanisms and cell damage [51].

5-FU-induced cardiotoxicity can be associated with free radical damage to the myocardium [1, 52] Drugs such as 5-FU can produce large amounts of free radicals, and the accumulation of these substances can lead to cytotoxicity, lipid peroxidation in membranes, and cell death [36]. Since the end product of cell membrane lipid peroxidation is MDA, its concentration indicates the severity of lipid peroxidation [53]. TAC, on the other hand, represents resistance to cell oxidative processes. Sengul et al. showed that 5-FU increased MDA levels and reduced the activities of superoxide dismutase (SOD) and glutathione (GSH) [28]. Therefore, in this study, we evaluated MDA and TAC levels to specify the role of oxidative stress in cardiotoxicity induced by 5-FU. The current study showed that administration of 5-FU increased MDA levels as well as decreasing TAC values [44]. On the other hand, administration of KPF reduced MDA levels in treatment groups; however, this reduction was not significant when we used KPF-NPs. TAC values also increased in treatment groups, but this increase was not significant compared to the 5-FU group. These results are aligned with previous reports [54] and the use of antioxidants such as KPF, which minimize reactive oxygen species and cell damage.

In addition, evaluating the histopathological changes in cardiac tissue supports the results obtained from biochemical analyzes.

A histopathological study of 5-FU-induced cardiotoxicity was performed on albino rats and multiple interstitial myocardial hemorrhages, inflammatory reactions, multifocal myofiber necrosis, vascular changes, pericarditis, and valvulitis were observed especially in the left ventricle [55]. The results of the histopathological analysis of our study indicated degenerative changes, including necrosis, hyaline formation, and hyperemia in rat cardiac tissue followed by 5-FU treatment. Treatment with KPF as well as KPF-NPs reduced cardiac tissue damage, including necrosis, hyaline formation, and hyperemia which could be attributed to the protective and anti-inflammatory effects of KPF. Our results are inconsistent with the previous finding which evaluated the effect of KPF on isoproterenol-induced injury to the myocardium and was shown that rats pretreated with KPF showed a reduction in the myocardial infarcted area [30]. Another study in this regard showed the positive effect of KPF on histopathological changes and heart failure in isoproterenol-induced heart failure in male diabetic rats [45].

Studies suggested KPF be useful for relieving inflammation [56]. It has been reported that KPF could possibly suppress COX-2 expression in lung tissue and as a beneficial anti-inflammatory agent against allergens, which reduces respiratory inflammatory reactions in the lungs and trachea [57]. Numerous studies have reported that KPF has a strong inhibition of COX 1 and 2 enzymes in the in vitro environment [58]. Lee et al. also showed that KPF could inhibit COX-2 expression by inhibiting Src-kinase activity [59]. The KPF treatment also decreased the COX-2 expression in cardiac tissue of isoproterenol-induced heart failure rats [45]. The COX-2 is a proinflammatory enzyme, and its expression is triggered by several stimulants such as hypoxia or free radical presence. Selective inhibitors of COX-2 have been used to ameliorate chronic and acute inflammation. COX-2 role in cardiac injury is controversial [60]. 5-FU resulted in increased expression of COX-2 in the heart tissue which led to elevated ROS production [61]. The doxorubicin-induced COX-2 expression in the cardiac tissue has been reported in different studies which could demonstrate COX-2 role in doxorubicin-induced cardiac damage [60]. Similarly, the findings of Ibrahim et al. and Delgado et al. showed that COX-2 inhibition ameliorated cardiotoxicity and heart failure induced by doxorubicin, respectively [60, 62]. On the other hand, some studies reported that increased COX-2 expression levels in cardiac tissue induced by doxorubicin, protected cardiac cells against apoptosis, and COX-2 inhibition were associated with exacerbation of cardiac injury [60]. Dowd et al. announced that inhibition of COX-2 deteriorated cardiac injury induced by doxorubicin [63]. Our results showed that the COX-2 expression decreased in treatment groups; however, this reduction was only significant in vitamin C and 1 mg KPF treatment groups. VEGF is an important factor in pathogenesis in response to stimuli and injuries and provides the ability to activate the signaling cascade and the ability of endothelial cells to migrate [64]. VEGF is an important proinflammatory cytokine that improves neovascularization in hearts of ischemic heart disease patients [65]. Application of VEGF-containing NPs increased angiogenesis as well as reducing adverse cardiac remodeling in mice with myocardial infarction [66]. VEGF-B upregulation induced by resveratrol improved ischemia/reperfusion injury in the myocardium of rats [67]. VEGF-A levels were increased in rats and were associated with microvessel density in the infarcted area [65]. We similarly found that KPF treatment significantly increased VEGF levels in the heart which could indicate KPF ameliorative role in ischemia/reperfusion injury induced by 5-FU. Although many studies have been conducted on the protective effects of some flavonoids on the cardiotoxicity of 5-FU, the present study is the first to demonstrate the cardiac protection effects of KPF and KPF-NPs against 5-FU-induced heart damage in rats.

Many studies have been done in this field. However, in the current study, despite the increased expression of COX-2 and VEGF in the 5-FU group, no significant difference was observed in the expression of COX-2 and VEGF genes in the groups treated with KPF and NPs compared to the 5-FU group.

## 5. Conclusion

In conclusion, the results of the present study showed that treatment with KPF and its NPs has approximately similar therapeutic effects on cardiac protection. KPF and KPF-NPs have also been shown to increase RBC, WBC, HB, and PLT levels. In addition, they reduce the level of serum enzymes in the heart. This study also indicated that KPF and NPs could protect the heart through biochemical and histological changes. These data could be helpful to better explore the benefits of KPF as a new treatment strategy for protecting the heart against chemotherapy drugs.

## Figures and Tables

**Table 1:** The utilized primer sequence.

Primer	5′----3′
COX-2 FW	CAACCAGCAGTTCCAGTATCAGA
COX-2 RV	CAAGGAGGATGGAGTTGTTGTAGAG
VEGF FW	TGTGGACTTGAGTTGGGAGGAGG
VEGF RV	GGCAGGCAAACAGACTTCGGC
GAPDH FW	CTACATGGCCTCCAAGGAGTAAG
GAPDH RV	CCTCCTCTTCTTCGTCTATGGC

**Table 2:** The effect of different treated groups on histopathological changes of heart tissue in rats and then comparisons of different groups with each other.

Groups	Histopathological changes		
	Hyaline	Necrosis	Hyperemia
Normal	**—**	**—**	**—**
5-FU	**+**	**++**	**++**
5-FU+10 mg vitamin C	**+**	**+**	**+**
5-FU+ 1 mg KPF	**—**	**+**	**+**
5-FU+ 1 mg KPF-NPs	**—**	**+**	**+**

## Data Availability

Upon request, data supporting the conclusion of our study are accessible by corresponding author.

## Supplementary Materials

Supplementary Materials We included the graphical abstract in the supporting files.
