# Supplementary figures and images for: Protective Effect of Kaempferol and Its Nanoparticles on 5-Fluorouracil-Induced Cardiotoxicity in Rats
Source: Biomed Res Int. 2022 Feb 13;2022:2273000. doi: 10.1155/2022/2273000 (PMC8858719; doi:10.1155/2022/2273000)

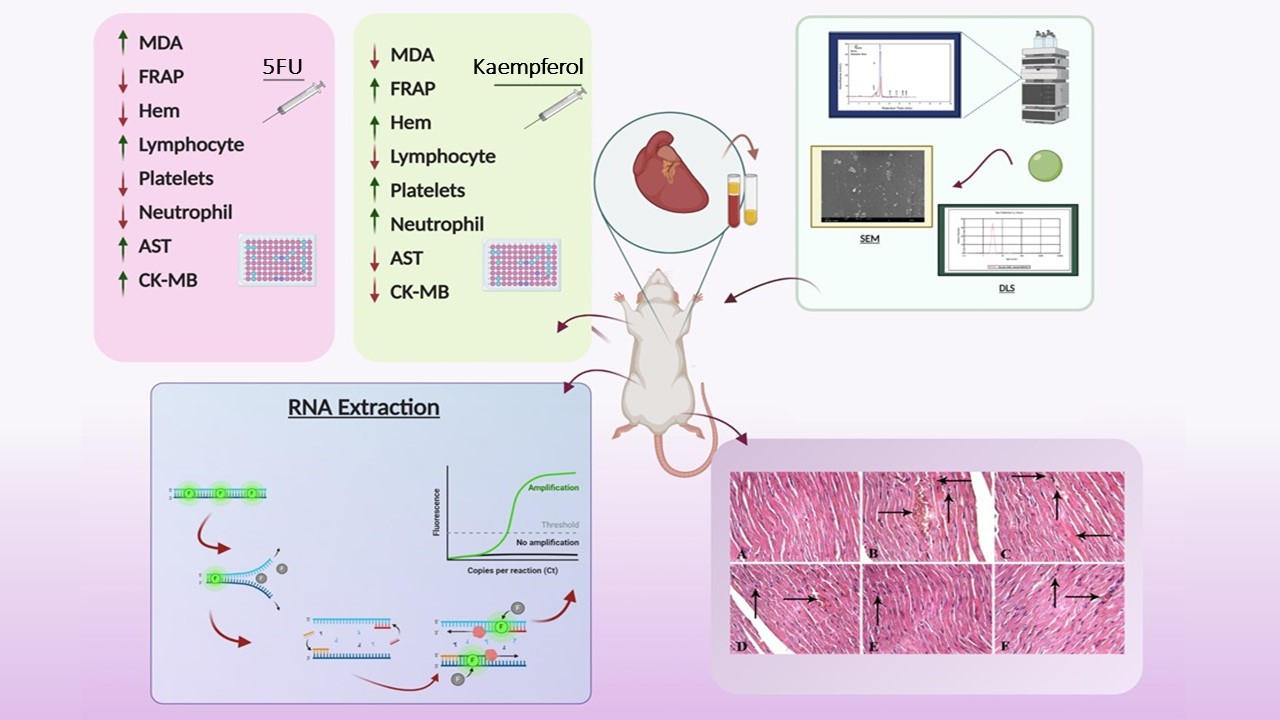

Supplement: Supplementary Materials — We included the graphical abstract in the supporting files. [file 2273000.f1.jpg]
